# Describing Pre–Post Changes Observed During the Implementation of a Snoezelen Program in a Real-School Context

**DOI:** 10.3390/ejihpe16050062

**Published:** 2026-04-30

**Authors:** María-Dolores Cárcel-López, Mercedes Ferrando-Prieto

**Affiliations:** 1 Department of Didacticsand School Organization, University of Murcia, 30100 Murcia, Spain; md.carcellopez@um.es; 2 AUFREN, 30107 Murcia, Spain; 3 Department of Developmental and Educational Psychology, University of Murcia, 30100 Murcia, Spain

**Keywords:** ASD, multisensory stimulation, sensory profile, repetitive behaviors, adaptation, educational intervention

## Abstract

Sensory alterations affect 90% of individuals with autism and have been recognized in the DSM-5 as a diagnostic criterion. These alterations often exacerbate emotional stress and may increase levels of anxiety, impacting everyday life activities. The general objective of this work is to assess the changes before and after a multisensory stimulation program aimed at improving sensory processing, repetitive behaviors, and adaptation to the environment in a sample of students with ASD. Twenty-seven schoolchildren participated (M = 10.04, SD = 4.24), with different levels of impairment, diagnosed by specialized teams. The design included a pretest and post-test condition. The results highlight significant improvements in the assessed areas. The Sensory Profile-2, the Bodfish Repetitive Behavior Scale, and the Vineland Scale were administered. The results suggest that changes may be conditioned by the student profile. The profiles that benefited the most in terms of sensory profile and repetitive behaviors were students with levels 1 and 3; meanwhile, students with level 2 did not show improvements in these areas but did show gains in overall adaptation, as measured by the Vineland Scale.

## 1. Introduction

Autism Spectrum Disorder (ASD) is a neurodevelopmental disorder characterized by deficits in communication and social interaction, as well as the presence of restricted and repetitive behavioral patterns (American Psychiatric Association, 2013). However, autism manifests differently in each individual, varying in the degree of impairment and intellectual and language development functioning, as well as depending on age. This perspective has driven a transition from a categorical approach to autism toward a dimensional perspective (Grosso Funes, 2021; Hervás Zúñiga et al., 2017). It was not until 2013 that one of the most characteristic experiences in individuals with ASD—difficulties in sensory processing—was recognized as a diagnostic criterion in the DSM-5.

### 1.1. Sensory Alterations in ASD

The sensory development of all individuals is the foundation of all knowledge. In the learning process, this development decisively influences the ability to process internal and environmental sensations, as well as the ability to make appropriate use of those sensations to plan and organize adaptive behavior (Lázaro & Berruezo, 2009). In 90% of individuals with ASD (Cañadas Pérez, 2021; Leekam et al., 2007), this process appears to be altered throughout the neurobiological pathway, from the registration of stimuli to the cortical integration of all sensations in order to generate an appropriate response in interactions with others and with the environment.

These alterations refer to the term sensory processing disorder (Miller et al., 2007), which is defined as difficulties in processing and using sensory information to regulate physiological, motor, affective, or attentional responses, interfering with the organization of behavior and the execution of activities of daily living (Miller et al., 2007, p. 93). These alterations can be classified into three major categories: sensory modulation disorders, sensory discrimination disorders, and sensory-based motor disorders. In addition, the classification proposed by Miller et al. (2007) differentiates according to stages of perceptual processing: sensory input, cortical processing, and motor output.

Sensory modulation problems refer to difficulties in transforming sensory information into an appropriate response to sensory stimuli, which may result in hyperreactivity, hyporeactivity, or sensation seeking (Iarocci & McDonald, 2006). Sensory hyperreactivity manifests itself when there is an exaggerated response to stimuli that, for most people, would be neutral or even pleasant (Bogdashina, 2007). For example, a child with ASD may respond with distress to bright lights, unexpected noises, or contact with certain textures (Dunn, 2014). This extreme sensitivity may lead to avoidance of certain situations, increasing social isolation and anxiety (Green & Ben-Sasson, 2010). In contrast, sensory hyporeactivity involves a diminished response to environmental stimuli, which may cause the child to fail to respond adequately to pain or to relevant auditory and visual signals (Cárcel-López, 2024). In such cases, children may appear disconnected from their environment, making it difficult for them to participate in educational and social activities (Tomchek & Dunn, 2007).

Difficulties in sensory discrimination affect an individual’s ability to distinguish between exteroceptive and interoceptive sensory stimuli (Pollock, 2009). This neurological disorganization can occur when: (a) the brain does not receive sensory stimuli due to a disconnection, (b) the brain receives incorrect sensory messages, or (c) the brain constantly receives sensory messages but does not properly connect them with other sensory messages to produce a meaningful response. For example, some children with ASD may have difficulty differentiating between similar sounds, which affects their understanding of spoken language and their ability to follow instructions in noisy environments (Cervera et al., 2014). Likewise, they may have difficulties identifying differences in textures or temperatures, which impacts their capacity to carry out daily tasks such as dressing or handling objects precisely (Angulo et al., 2020).

Finally, sensory-based motor disorder is related to the integration of sensory information to coordinate movement, and refers to the difficulty in stabilizing the body during movement or at rest in order to meet the demands of both the environment and a given motor task (Cárcel-López, 2024). Many children with ASD display motor planning difficulties, which can affect their ability to perform daily activities such as dressing, eating, or writing (Ayres, 1979). These difficulties may be related to alterations in neural connectivity that affect how the brain processes and responds to sensory information (Cárcel-López, 2024).

Sensory alterations in individuals with ASD significantly impact their daily lives. Often, the most basic home routines, such as getting up, showering, or eating (Grandin, 2019), become a major effort. In addition, these alterations may reduce participation in various daily and leisure activities such as going to the supermarket, school, cinema, park, or dentist, where environmental noise or intense lighting can trigger anxiety processes in individuals with ASD (Hochhauser & Engel-Yeger, 2010). In other cases, feeding problems may arise due to aversion to textures (Zobel-Lachiusa et al., 2015), as well as emotional, academic, and behavioral difficulties in the school setting (González Alba & Ruiz Ariza, 2021). Sensory alterations have been linked to the repetitive behaviors characteristic of ASD (Cárcel-López & Ferrando-Prieto, 2025; Fetta et al., 2021; Noda et al., 2024); in this regard, a relationship has been suggested with certain self-injurious behaviors (Paula Pérez & Artigas, 2016). On the other hand, behavioral problems tend to manifest over time, with a prevalence ranging from 57% to 90% (Kanne & Mazurek, 2011). Aggressiveness is one of the most common problems, affecting 69% of cases against caregivers and 49% against people outside their closest circle (Allen et al., 2008). Temple Grandin points out that, through the proper use of sensory channels and appropriate sensory therapeutic intervention, it is possible to successfully address highly dramatic situations such as aggressiveness.

### 1.2. Sensory-Based Therapy

For decades, occupational therapy has proposed interventions to support sensory processing. In this regard, Ayres, a pioneer in the field, emphasized the need to work on Sensory Integration (SI) as an approach to improve the organization of sensations in the central nervous system and their use in daily life. According to current knowledge, four major approaches to sensory intervention can be distinguished: (1) Ayres Sensory Integration; (2) the Senses-based intervention approach; (3) Sensory diets; (4) Multisensory stimulation through Snoezelen environments. A comparison of these methodologies following Cárcel-López (2024) can be found in Table 1.

This intervention was selected based on the following criteria:

(a) Sensory diets focus mainly on self-regulation, which largely depends on both cognitive abilities and the individual’s age; hence, they would not be effective for students with severe impairments who may also present comorbidities, including intellectual disability.
(b) The direction of the session: In sensory integration therapies, the session is directed by the student (the individual directs their own actions while the therapist manages the environment); however, we believe that for individuals with ASD, structured spaces and times work much better.
(c) Additionally, we consider it preferable to use a multisensory approach; therefore, sense-based therapy, which predominantly uses monosensory strategies, was excluded. People with autism have altered processing in multiple senses simultaneously; consequently, the most appropriate practice would be to apply evidence-based multisensory stimulation to optimize outcomes.

### 1.3. Research on Multisensory Stimulation Rooms

Although many reviews have been published on the efficacy of sensory interventions (e.g., Camino-Alarcón et al., 2024; Schoen et al., 2019), few focus specifically on the Snoezelen methodology. In this regard, the review by Cárcel-López (2024) provides a broad overview of the state of the art: most research has concentrated on analyzing the impact of this intervention on reducing stereotypies, aggression, self-injury, anxiety, and, more generally, disruptive and maladaptive behaviors in individuals with autism (e.g., Bova et al., 2018; Novakovic et al., 2019; Unwin et al., 2022). A smaller number of studies have focused on improvements in sensory processing itself (e.g., Derakhshanrad & Piven, 2020; Habbak & Khodeir, 2023) and enhancements in communication and interaction with the environment (e.g., B. Pfeiffer et al., 2017; Teodoro et al., 2018), as well as improvements in attention, wellbeing, meaningful learning, and mood (e.g., Soltani Taleghani et al., 2021; Unwin et al., 2022). In nearly all cases, the intervention results were promising.

Its integration into educational and therapeutic contexts represents a promising strategy to improve the quality of life and wellbeing of these individuals, promoting their active participation in various learning and socialization environments.

Despite the growing popularity of multisensory interventions, existing empirical studies present substantial methodological limitations, including pre–post designs without control groups, small samples, reliance on caregiver reports, and inconsistent outcome measures (Case-Smith et al., 2015; Maskey et al., 2019; Leonardi et al., 2025). The studies of Novakovic et al. (2019), Soltani Taleghani et al. (2021), and Swathi (2016) are an exception.

In most cases, the samples in previous studies were focused on levels 2 and 3 of ASD. Studies with mixed samples do not specify subtypes. As the spectrum is quite diverse, it is important to understand which students’ profiles can benefit more from this intervention. Most multisensory stimulation studies have been conducted in highly structured residential, therapeutic, or special needs school settings, where Snoezelen rooms are embedded in institutional care routines rather than in ordinary public school classrooms. This makes it especially relevant to examine how a multisensory program operates within a mainstream Spanish school context, under real-world organizational constraints. These limitations restrict causal inference and highlight the need for cautious interpretation of reported improvements.

In this context, the present study seeks to contribute to the psychoeducational field by adopting an exploratory, practice-based approach. Rather than testing causal effectiveness, it aims to document patterns of change^1^ associated with the implementation of a multisensory stimulation program in a real-world school context.

The following specific objectives were established:

1. To evaluate changes before and after the program in three aspects: sensory processing, repetitive behaviors, and adaptation.
2. To examine the relationship between improvements in sensory processing and changes in repetitive behaviors and adaptation among the participants.

The Spanish educational context provides a relevant setting for examining multisensory interventions, as Snoezelen rooms are increasingly implemented in publicly funded schools despite limited empirical evaluation within this system. Educational inclusion policies, the widespread use of specialized open classrooms, and differences in service provision compared to Anglo-Saxon contexts warrant context-specific exploratory research.

## 2. Materials and Methods

### 2.1. Participants

The study was conducted in a publicly funded mainstream school with a strong policy on integration. The school invested in a Snoezelen-based multisensory room as part of its inclusive education policy. Once the room was installed, the school leadership decided that all eligible students with ASD should have access to this resource as part of their usual support plan.

In this context, the creation of a no-treatment control group was considered both ethically and practically unfeasible, as it would have involved depriving some families of an intervention that the school had already incorporated into its provision. For this reason, a pre-experimental one-group pretest–post-test design was adopted, focusing on documenting patterns of change under real-world conditions rather than establishing causal efficacy.

The initial sample was composed of 30 students. But due to experimental death (change of school, unable to take them to the stimulation room), the final sample consisted of 27 schoolchildren (M = 10.04; SD = 4.24) from an educational center in the Region of Murcia. Table A1 shows the details of the participants. All the students had a diagnosis of ASD with multiple disabilities and were diagnosed by the Specialized Educational and Psychopedagogical Guidance Team for Autism and other Severe Developmental Disorders of the Department of Education. Fifteen students were enrolled in mainstream integration (regular setting with support), and 15 in Specialized Open Classrooms (for students with extensive support needs). Of the total sample, 7 schoolchildren were diagnosed with Level 1 ASD, while 20 presented comorbidity with intellectual disability, placing them at Level 2 or 3 of the autism spectrum (8 in Level 2 and 12 in Level 3).

The wide age range and functional heterogeneity reflect the ecological reality of specialized educational settings for students with ASD and intellectual disability. Given the exploratory and practice-based nature of the study, this heterogeneity was retained to examine whether patterns of change differed according to ASD severity levels rather than age-based grouping.

### 2.2. Instruments

In the pretest and post-test phases, the instruments were completed by the same informants. Specifically, the classroom teachers of the participating students, as well as the educators who supported the teacher in cases where students were enrolled in specialized open classrooms, filled out the measures at both the beginning and the end of the program.

Measurement of sensory processing. The Spanish version of Sensory Profile-2 by Dunn (2014) was used. The instrument was validated by Pearson Educación (Dunn, 2016) using a Spanish sample of 621 students. This instrument assesses sensory responses in both children and adults. It evaluates 86 behaviors related to sensory sensitivity across different modalities: auditory, visual, tactile, and other sensory areas. It is based on Dunn’s Sensory Processing Model, which classifies sensory responses into four main patterns: seekers, avoiders, sensors, and bystanders. According to the Spanish version, the internal reliability of the scale ranges from 0.72 to 0.90.

Although the Sensory Profile-2 was originally structured according to Dunn’s four sensory patterns, for the purposes of this study, we created two composite indices reflecting sensory hyperreactivity and hyporeactivity, in line with current autism research on sensory modulation. These composites were obtained by summing relevant subscales and were used as exploratory indicators of sensory reactivity, not as standardized test scores. Therefore, hyperreactivity was composed of the Sensitivity and Avoidance patterns, whereas hyperreactivity was composed of the Registration pattern.

For the purposes of this study, scores from the Sensory Profile-2 were grouped into two exploratory composite indices: Hyperreactivity (integrating the Sensitivity and Avoiding quadrants) and Hyporeactivity (based on the Registration quadrant). This methodological decision is grounded in the sensory modulation literature, which identifies the neurological threshold as the determining factor of reactivity (Baranek et al., 2019; Boyd et al., 2010) and is consistent with DSM-5 diagnostic criteria, which prioritize the distinction between excessive and diminished responses.

The Sensory Seeking quadrant was excluded, given that although it shares a high threshold with hyporeactivity, its active behavioral manifestation represents a qualitatively distinct stimulus-approach construct in the ASD population (Ben-Sasson et al., 2009).

It is important to note that these indices are exploratory composite scores and do not replace the test’s standard scores. This pragmatic restructuring aims to enhance the interpretability of the results in the context of sensory modulation. However, it does not constitute a formal validation of the construct; therefore, future research should confirm this structure using confirmatory factor analysis or Rasch models in larger samples.

Repetitive behaviors were measured using the Repetitive Behavior Scale-Revised (RBS-R) (Bodfish et al., 1999). Composed of 43 items, this scale assesses stereotyped, compulsive, self-injurious, and ritualistic behaviors using a 4-point Likert scale. The scale has been adapted to the Spanish context and has shown strong psychometric properties, with an α = 0.97 for the total scale and test–retest ICC indices between 0.97 and 0.98 (Martínez-González & Piqueras, 2018). Since the literature distinguishes between two main types of repetitive behaviors (sensory–motor vs. insistence on sameness) (Noda et al., 2024), a principal component analysis with varimax rotation was conducted on the subscales, forcing a two-factor solution. According to this analysis, the first component consisted of stereotyped, self-injurious, and compulsive behaviors (corresponding to motor behaviors), while the second component included ritualistic, perseverative, and sameness behaviors (corresponding to insistence on sameness). The sensory–motor component explained 45% of the variance, and insistence on sameness explained 24.5% of the variance.

Adaptive behaviors were measured through the items of the Vineland-3 Adaptive Behavior Scales (Sparrow et al., 2016).

Adaptive functioning was assessed with the Vineland-3 Adaptive Behavior Scales (Sparrow et al., 2016), an internationally validated measure widely used in autism and intellectual disability research. Although a formally adapted Spanish version is not yet available, the instrument was chosen because it provides a comprehensive assessment of communication, daily living skills, and socialization, which are central outcomes in school-based interventions.

This scale is designed to assess the development of adaptive skills in the areas of communication, daily living, social skills, and motor activity. It consists of 433 items distributed across these dimensions, which are rated on a 3-point Likert scale (0: never, 1: sometimes, 2: usually), allowing for the measurement of the degree of autonomy and social integration of the individual. The scores are transformed into standardized scores to interpret the level of adaptive development. In our study, we decided to use the raw scores of each subscale, since we are more interested in examining the individual progress of each student rather than comparing them with their age group, given the particular characteristics of the sample. Raw scores were used to capture absolute within-individual change over time rather than age-normed comparisons, which may obscure small but meaningful gains in populations with severe developmental delays. The CI scores can be consulted in Table A2.

### 2.3. Procedure

Following approval by the Ethics Committee of the University of Murcia, the management team of a state-subsidized school in the Region of Murcia was approached to carry out a study in which a multisensory stimulation program was implemented with the aim of evaluating its impact on participants’ sensory and behavioral regulation. With the commitment of the school leadership and the informed consent of the participating families, the sample was selected and assessed.

Based on the results of the initial evaluation and the scientific literature, a structured intervention program was designed, targeting motor, cognitive, and sensory regulation skills. For its implementation, a Snoezelen room was equipped with specific materials for visual, auditory, tactile, and vestibular stimulation.

The intervention was implemented during the 2018–2019 academic year, consisting of 28 individual sessions per student (once a week over seven months, from December to June). An initial assessment was conducted to establish each student’s sensory profile. This information was used to tailor the intervention to each participant.

The staff responsible for both the intervention and the evaluation in the multisensory stimulation classroom were selected as the students’ primary educators and received specific training prior to the start of the study. The selected teachers each had over 10 years of experience at the center and were highly familiar with teaching and learning strategies for students with ASD. Each teacher worked with 2 or 3 students in one-on-one sessions. A total of 15 educators participated in the program implementation (60% female, 40% male), with a mean age of 33.4 years (SD = 3.2).

It is important to note that several adaptations were required when applying this methodology to individuals with ASD, involving a shift in the underlying therapeutic paradigm. Whereas in traditional Snoezelen rooms the central focus is the relationship between therapist and client, in our case, the intervention was organized around a reference person, and all sensory stimulation was delivered while explicitly avoiding physical contact. In Table A3, we expose the application protocol used.

The elements of the multisensory room were therefore used in different ways, with distinct objectives depending on the child’s level of autism severity. For students at level 3, the primary focus was on developing proprioceptive and vestibular skills; for those at level 2, visual, auditory, and other sensory modalities were additionally targeted; and for students at level 1, it was possible to formulate goals oriented toward the enhancement of logical and critical thinking (see Table A4). Further details on the intervention plan can be found in (Cárcel-López, 2024).

### 2.4. Data Analysis

A descriptive analysis of the data (means, standard deviations) was conducted, as well as group comparisons using the Wilcoxon test, given the sample size and the non-normal distribution of the data. Correlation analyses were also performed using Kendall’s tau_b test. The data were analyzed using the statistical package SPSS v.26 for Windows.

## 3. Results

First, descriptive statistics were calculated for each variable for the total sample and for each group of students according to their level of ASD severity (Table 2). Looking at the overall group of participants, we can see that in the scores for sensory processing, there was a statistically significant improvement using the Bonferroni adjustment: higher hyperreactivity decreased to lower hyperreactivity, and similarly, higher hyporeactivity decreased to lower hyporeactivity; these changes were statistically significant (both with a moderate size effect of about 0.6). Therefore, for the overall sample, there was an improvement in sensory processing. Repetitive behaviors (motor and sameness) appeared to remain stable before and after the program. Adaptive skills (communication, daily living skills, and motor skills) also improved for the total sample.

Given the heterogeneity of the participants, changes were analyzed considering the ASD severity level of the students. Since the program could show different effectiveness depending on the students’ profiles, mean comparison tests were conducted for each severity level (Level 1, Level 2, and Level 3).

It was observed that students at Level 3 benefited from the implementation of the program and improved their sensory processing (showing reductions in both hyperreactivity and hyporeactivity).

Regarding repetitive behaviors, no statistically significant differences were found between the pre- and post-test, not for the overall sample nor for any of the groups by autism level.

When examining adaptive scores by group of students, none of the groups reported statistically significant improvement. Only the Level 2 group showed a statistically significant improvement for daily living skills (Z = −2.371 d; *p* = 0.018; effect size = 0.84).

Given the limited sample size, regression analyses were ruled out. Instead, correlations between the variations in each variable were examined. To this end, the “gain” (or loss) in each variable was calculated. Table 3 shows Kendall’s tau_b correlations for the difference scores between pretest and post-test conditions in each variable.

It was observed that most statistically significant correlations occurred between variables belonging to the same construct. Thus, the two sensory processing variables—hyperreactivity and hyporeactivity—showed a correlation (τ_b = 0.44, *p* < 0.01). Similarly, the two repetitive behavior variables showed a correlation (τ_b = 0.45, *p* < 0.01). The only statistically significant cross-construct correlations were found between motor repetitive behaviors and communication (τ_b = −0.33, *p* = 0.027), and between motor repetitive behaviors and daily living skills (τ_b = −0.35, *p* = 0.018), both negative. In other words, higher motor repetitive behavior was associated with lower communication and fewer daily living skills.

## 4. Discussion and Conclusions

This study aimed to empirically analyze the changes after an educational intervention based on the Snoezelen approach, directed at students with Autism Spectrum Disorder (ASD). Although the use of multisensory rooms is common in educational and therapeutic settings, the scientific literature indicates that empirical evidence regarding their actual impact remains limited and sometimes controversial (Leonardi et al., 2025).

ASD is a complex and heterogeneous neurobiological condition that poses significant challenges for applied research. In our study, methodological limitations stand out, such as a small sample size and the absence of a control group—common difficulties in research conducted in real school settings. These limitations should be taken into account when interpreting the results, as they may influence the generalizability and robustness of the findings.

Despite these constraints, after the Snoezelen intervention, statistically significant improvements were found in participants’ sensory profiles, both in hyperreactivity and hyporeactivity domains. It should be noted that the research design does not allow these changes to be attributed to the program or to rule out other explanations. It is noteworthy that few studies on sensory intervention have measured the effects on the sensory processing profile. B. A. Pfeiffer et al. (2011), who did measure this, found no significant changes in sensory processing before and after the program.

Statistically significant improvements were also observed in the ‘adaptation’ domain. These results align with existing literature indicating that targeted sensory processing interventions support self-regulation and wellbeing in individuals with Autism Spectrum Disorder (ASD) (B. A. Pfeiffer et al., 2011; Schaaf et al., 2014). For instance, Schaaf et al. (2014) reported significant gains in self-care, as measured by the PEDI scale, noting a reduction in the level of assistance required from caregivers. These findings are further supported by Teodoro et al. (2018), whose case study confirmed improvements in communication and task comprehension following a Snoezelen intervention.

Furthermore, the correlation between sensory processing—particularly auditory and tactile—and social interaction challenges has been explicitly established (Derakhshanrad et al., 2024). When comparing ABA, Snoezelen, and Dosa methods, Soltani Taleghani et al. (2021) observed significant improvements in social skills and attention across all groups. This suggests that while various interventions yield positive outcomes, such gains could also be influenced by underlying maturational processes.

Regarding our own data, a comparison of pretest and post-test raw scores indicates more pronounced changes in students requiring lower levels of support. In contrast, students with Level 3 Autism demonstrated a notably slower rate of developmental progress.

No statistically significant changes were observed regarding repetitive behaviors following the program’s implementation. This dimension remains one of the most debated in the literature, with inconclusive results across various studies (Cárcel-López & Ferrando-Prieto, 2025). For instance, while Novakovic et al. (2019) reported a significant decrease in autism severity and repetitive behaviors in a sample of 40 adolescents and adults, other studies offer a more cautious perspective. Bova et al. (2018) found no consistent evidence of such reductions in adults, noting highly individualized responses. Similarly, McKee et al. (2007) observed that none of their participants showed a decrease in disruptive behaviors outside the intervention setting, with one participant even demonstrating an increase during the sessions.

Although previous research (B. A. Pfeiffer et al., 2011; Schaaf et al., 2014; Case-Smith et al., 2015) suggests moderate improvements in self-regulation and problematic behaviors, these effects are often short-term and difficult to generalize without family-based or contextual support. The lack of significance in our findings may be attributed to the intervention protocol. As noted by Case-Smith et al. (2015), sensory interventions are most effective when implemented in response to the child’s immediate arousal state, rather than through a fixed once-a-day schedule. Furthermore, it has been suggested that significant behavioral changes may require higher session intensity than what was provided in this study (Fava & Strauss, 2010). It is important to highlight that repetitive behaviors in ASD may serve an adaptive function as mechanisms of emotional and sensory self-regulation (Joyce et al., 2017). Therefore, an increase in these behaviors should not always be interpreted as a sign of deterioration, but possibly as a functional response to the environment.

We also examined whether outcomes varied according to ASD severity levels, following Bova et al. (2018), who suggested that systematic improvements in repetitive behaviors are more prevalent in individuals with milder impairments. While our decision to stratify the sample was clinically motivated, no substantial differences between profiles were observed. However, it is important to note that while effect sizes within certain severity levels were large, the combination of small sub-group samples and conservative corrections for multiple comparisons limited the statistical power to detect significance. Conversely, the analysis of the total sample—which integrated consistent patterns of change, particularly in levels 1 and 3—reached significance with moderate effect sizes. This suggests that the lack of significance in stratified analyses may stem from power constraints rather than an absence of clinically relevant change. These constraints, along with the inability to analyze variables such as age, represent important limitations to be addressed in future research with larger cohorts.

The correlational analysis of pre–post changes across variables reveals that improvement in the sensory profile is not necessarily associated with a reduction in repetitive behaviors or increased functional adaptation. Only motor repetitive behaviors showed a significant correlation with improvements in communication and daily living skills, suggesting that a reduction in these behaviors might indirectly facilitate functional participation, in line with findings by Case-Smith et al. (2015).

Overall, these results suggest that the impact of the Snoezelen intervention may be neither linear nor homogeneous, but appears to occur through a chain of indirect and mediated effects that may not be evident in simple or short-term studies. To unravel the mechanisms of action and timing of effects, it is essential to develop future research using more robust methodological designs, including control groups and longitudinal follow-up (Kasari et al., 2012). Only in this way will it be possible to determine the true potential and limitations of the Snoezelen approach in interventions with students with ASD.

Recent systematic reviews (e.g., Case-Smith et al., 2015; Leonardi et al., 2025) and meta-analyses report that many studies present a risk of bias due to small sample sizes, absence of control groups, and lack of longitudinal follow-up. Emphasis is placed on improving methodological quality by using randomized designs and standardized pre–post intervention measures. As is the case with our study, some of these limitations are highlighted, such as the lack of a control group and the use of information depending on teachers’ observations, which may reduce objectivity.

## Figures and Tables

**Table 1:** Comparison of sensory intervention approaches.

Characteristic	Ayres Sensory Integration	Sensory-Based Intervention	Sensory Diets	Multisensory Stimulation (Snoezelen)
Definition	Clinical method to improve the brain’s ability to organize and process sensory information.	Interventions aimed at stimulating one or more senses to improve sensory responses.	Personalized strategies provide specific sensory stimuli throughout the day.	Stimulation of multiple senses in controlled environments designed to promote wellbeing, relaxation, or activation through sensory input.
Underlying Principle	Jean Ayres’ Sensory Integration Theory.	Stimulation of individual or combined senses.	Individual sensory needs with a daily focus.	Immersive multisensory experience without requiring a rigid structure.
Main Goals	Improve sensory discrimination, sensory modulation, and praxis.	Improve specific responses to sensory stimuli.	Regulate the child’s level of arousal and responses throughout the day.	Provide wellbeing, relaxation, cognitive and sensory stimulation, or activation.
Clinical Application	Structured programs conducted by occupational therapists.	Used by therapists, teachers, or caregivers.	Guided by therapists but applied in various settings (home, school, etc.).	Used in Snoezelen rooms specifically designed by teachers or therapists.
Type of Intervention	Individualized, structured, and based on clinical assessment.	Can be structured or flexible depending on the goal.	Personalized strategies integrated into daily routines.	Users explore freely, although it can also be used in a planned manner.
Setting	Specialized clinics or adapted areas.	Various settings (therapy, home, school).	Everyday environments such as home and school.	Snoezelen rooms (controlled and prepared environments).
Examples of Activities	Swinging, textured play, deep pressure, etc.	Play with lights, soft music, and specific textures.	Use of weighted balls, chew toys, rocking equipment, etc.	Play with lights, relaxing sounds, ball pools, scents, etc.

**Table 2:** Descriptive statistics of pretest and post-test scores and mean comparisons for the total sample and for each group.

		Pretest					Post-Test					Comparations	
		Range	Min–Max	M (SD)	Skew-Ness	Kurt-Osis	Range	Min–Max	M (SD)	Skew-Ness	Kurt-Osis.	(Wilcoxon) a	Effect Size
Hyperreactivity	LEVEL 1	101	44–145	75.86 (33.56)	1.73	3.53	29	28–57	38.86 (10.68)	0.66	−0.37	Z = −2.366 b; *p* = 0.018	0.89
	LEVEL 2	47	28–75	48.88 (14.83)	0.43	0.13	49	34–83	53.38 (14.74)	1.01	1.92	Z = −1.332 d; *p* = 0.183	0.47
	LEVEL 3	53	45–98	81.17 (16.09)	−1.15	0.74	53	37–90	58.17 (16.54)	0.76	−0.4	Z = −2.907 b; *p* = 0.004	0.84
	ALL	117	28–145	70.22 (25.15)	0.76	1.48	62	28–90	51.74 (16.28)	0.76	0.22	**Z = −3.437 b; *p* = 0.001**	0.66
Hyporeactivity	LEVEL 1	25	20–45	36.14 (10.81)	−1.14	−0.89	28	13–41	22.86 (8.93)	1.58	3.29	Z = −2.366 b; *p* = 0.018	0.89
	LEVEL 2	31	14–45	29.38 (10.04)	0.24	−0.28	19	18–37	28.50 (7.03)	−0.26	−1.39	Z = −0.070 d; *p* = 0.944	0.02
	LEVEL 3	22	42–64	49.67 (6.64)	1.03	0.62	33	23–56	40.25 (10.60)	−0.45	−0.66	Z = −2.667 b; *p* = 0.008	0.77
	ALL	50	14–64	40.15 (12.43)	−0.43	−0.32	43	13–56	32.26 (11.70)	0.3	−0.93	**Z = −3.342 b; *p* = 0.001**	0.64
Motor Repetitive Behaviors	LEVEL 1	25	0–25	9.71 (9.03)	0.82	−0.29	7	0–7	2.43 (2.64)	1.03	−0.05	Z = −1.693 b; *p* = 0.09	0.64
	LEVEL 2	8	2–10	5.50 (2.93)	0.34	−0.86	28	0–28	10.25 (8.35)	1.36	3.1	Z = −1.352 d; *p* = 0.176	0.48
	LEVEL 3	34	1–35	16.09 (11.12)	0.7	−0.21	22	4–26	16.08 (6.44)	−0.06	−0.33	Z = −0.267 d; *p* = 0.79	0.08
	ALL	35	0–35	11.12 (9.64)	1.25	1.08	28	0–28	10.81 (8.35)	0.5	−0.62	Z = −0.135 c; *p* = 0.893	0.03
Repetitive Behaviors Related to Sameness	LEVEL 1	38	1–39	18.00 (14.69)	0.29	−1.6	3	1–4	2.43 (1.13)	−0.24	−1.23	Z = −2.043 b; *p* = 0.041	0.77
	LEVEL 2	16	0–16	8.75 (6.48)	−0.26	−1.79	26	0–26	14.88 (8.72)	−0.38	−0.46	Z = −2.201 c; *p* = 0.028	0.78
	LEVEL 3	43	0–43	12.55 (12.78)	1.31	2.33	28	0–28	16.73 (8.06)	−0.75	0.69	Z = −1.021 c; *p* = 0.307	0.29
	ALL	43	0–43	12.85 (11.90)	1.06	0.77	28	0–28	12.31 (9.26)	0.13	−1.39	Z = −0.304 c; *p* = 0.761	0.06
Adaptation: Communication	LEVEL 1	76	172–248	201.00 (28.11)	0.99	0.36	76	172–248	217.17 (26.16)	−0.98	1.42	Z = −1.604 d; *p* = 0.109	0.61
	LEVEL 2	163	60–223	158.75 (57.62)	−0.77	−0.75	180	61–241	172.00 (59.52)	−0.97	0.24	Z = −2.313 d; *p* = 0.021	0.82
	LEVEL 3	79	20–99	52.27 (26.15)	0.7	−0.56	88	27–115	63.00 (28.22)	0.57	−0.27	Z = −2.028 d; *p* = 0.043	0.59
	ALL	228	20–248	122.04 (75.21)	0.12	−1.62	221	27–248	137.88 (77.60)	−0.02	−1.69	**Z = −3.463 c; *p* = 0.001**	0.67
Adaptation: Daily Living Skills	LEVEL 1	160	83–243	140.00 (67.68)	0.81	−1.21	176	78–254	163.33 (66.79)	0.26	−1.23	Z = −1.753 d; *p* = 0.08	0.66
	LEVEL 2	129	25–154	109.13 (46.24)	−0.9	−0.29	168	25–193	130.13 (54.23)	−1.07	0.9	Z = −2.371 d; *p* = 0.018	0.84
	LEVEL 3	38	32–70	47.18 (12.17)	0.76	−0.3	67	38–105	55.30 (21.64)	1.71	2.41	Z = −1.680 d; *p* = 0.093	0.48
	ALL	218	25–243	89.28 (56.80)	1.14	0.82	229	25–254	107.25 (64.99)	0.65	−0.48	**Z = −3.417 c; *p* = 0.001**	0.66
Adaptation: Socialization	LEVEL 1	138	63–201	116.67 (55.15)	0.82	−1.04	142	75–217	126.50 (47.52)	1.67	3.94	Z = −1.604 d; *p* = 0.109	0.61
	LEVEL 2	153	32–185	73.63 (49.03)	2.06	4.51	152	32–184	83.13 (47.74)	1.49	2.52	Z = −1.753 d; *p* = 0.08	0.62
	LEVEL 3	45	15–60	28.00 (12.94)	1.69	3.15	43	17–60	31.00 (13.33)	1.3	1.27	Z = −1.781 d; *p* = 0.075	0.51
	ALL	186	15–201	63.88 (52.18)	1.55	1.73	200	17–217	72.25 (52.94)	1.25	1.3	Z = −3.013 c; *p* = 0.003	0.58
Adaptation: Motor Skills	LEVEL 1	111	43–154	113.67 (40.63)	−1.11	1.22	100	54–154	124.17 (36.99)	−1.75	3.35	Z = −1.826 d; *p* = 0.068	0.69
	LEVEL 2	80	69–149	125.63 (29.49)	−1.34	0.61	75	77–152	136.13 (24.64)	−2.51	6.6	Z = −2.032 d; *p* = 0.042	0.72
	LEVEL 3	37	69–106	87.45 (11.79)	0.15	−1.01	38	68–106	90.30 (13.06)	−0.26	−1.04	Z = −1.405 d; *p* = 0.16	0.41
	ALL	111	43–154	105.96 (30.92)	0.06	−0.96	100	54–154	114.04 (31.47)	−0.24	−1.33	**Z = −3.182 c; *p* = 0.001**	0.61

a Wilcoxon Signed Ranks Test; b Based on positive ranks; c Based on negative ranks; d Subindex is used when “equal ranks”. Bonferroni-adjusted significance level for each analysis dimension (alpha = 0.0125). Bonferroni-adjusted significance level for 32 comparisons (alpha = 0.0015). Only *p* values ≤ 0.0015 are considered statistically significant. Effect sizes were calculated as $r=\frac{Z}{\sqrt{N}}$.

**Table 3:** Correlations between pre- and post-test difference scores for the various variables.

	1.	2.	3.	4.	5.	6.	7.	8.
1. Hyperreactivity	1							
2. Hyporeactivity	0.441 **	1						
3. Motor repetitive B.	0.102	0.109	1					
4. Sameness repetitive B.	0.163	0.1	0.454 **	1				
5. Adaptation: communication	−0.016	0.004	−0.336 *	−0.164	1			
6. Adaptation: daily skills	0.136	0.036	−0.356 *	−0.105	0.675 **	1		
7. Adaptation: socialization	0.042	−0.11	−0.148	0	0.223	0.348 *	1	
8. Adaptation: motor skills	−0.004	−0.105	−0.194	−0.131	0.337 *	0.308 *	0.304	1

Note: variable scores were computed by calculating the difference between pre- and post-test. * significance level at *p* < 0.05; ** significance level at *p* < 0.001.

## Data Availability

Data are available upon request.

## Note

1 By pattern of chance, we refer to the descriptive combinations of pre–post differences across sensory, behavioral, and adaptive domains that appear to vary by ASD severity level.

## Abbreviations

The following abbreviations are used in this manuscript:

ASD	Autism Spectrum Disorder

## Appendix A

**Table A1:** Descripción de los participantes en este estudio.

ID	Gender	Categoría Dimensional Del ASD Level *	AGE (Years)	Schooling
LQB	Woman	2	6	AA
SCL	Man	2	7	AA
MSA	Man	2	12	AA
VSL	Man	2	14	AA
LAS	Woman	2	15	AA
MMA	Man	2	16	AA
NMR	Man	2	16	AA
BFA	Woman	2	17	AA
MOVM	Woman	3	3	AA
CAC	Man	3	5	AA
MLP	Man	3	5	AA
RSJF	Man	3	7	AA
AMJ	Man	3	8	AA
RGJ	Man	3	9	AA
VME	Man	3	10	AA
VMT	Man	3	10	AA
TPL	Woman	3	14	AA
MLM	Man	3	14	AA
NGA	Woman	3	15	AA
OMJ	Man	3	18	AA
TMPC	Man	1	6	3º EI
SGA	Man	1	7	2º EP
ZSAJ	Man	1	7	2º EP
SPM	Man	1	8	3º EP
AAS	Man	1	9	4º EP
ZSF	Man	1	10	5º EP
CRJ	Man	1	13	2º ESO

(*) The severity levels proposed by the DSM-5 were used. AA: Open Classroom; EI: Early Childhood Education; EP: Primary Education. Level 3: “Requires very substantial support.” These individuals have minimal social communication; their behaviors are characterized by marked interference in daily life due to inflexibility and difficulties with shifting attention. Level 2: “Requires substantial support.” Their social communication shows a marked deficit, with limited initiation or reduced/atypical responses; their behavior frequently interferes with daily life due to inflexibility and difficulties with shifting attention. Level 1: “Requires support.” They can carry out social communication without on-site support, although they present significant impairments in social communication; their behavior shows unusual or excessive interest, but it does not interfere with daily functioning.

**Table A2:** Scores on Vineland Dimensions Using CI Scores Before and After the Program.

		Pretest			Post-Test		
	Dimension	Mean	Std. Deviation	Std. Error Mean	Mean	Std. Deviation	Std. Error Mean
ASD Level 1	Communication (CI score)	86.00	14,142	6325	86.60	13,107	5862
	Daily life skills (CI score)	75.20	16,392	7331	80.40	20,070	8976
	Socialization (CI score)	68.00	14,832	6633	73.80	20,030	8958
	Motor skills (CI score)	53.00	24,042	17,000	56.00	28,284	20,000
	VIN18_ABC_(total ci score)	76.20	14,550	6507	79.40	15,518	6940
ASD Level 2	Communication (CI score)	67.50	25,685	9081	63.75	22,474	7946
	Daily life skills (CI score)	62.13	20,441	7227	56.13	14,721	5205
	Socialization (CI score)	50.50	21,686	7667	48.00	16,222	5735
	Motor skills (CI score)	59.50	12,021	8500	64.50	23,335	16,500
	VIN18_ABC_(total ci score)	62.63	19,227	6798	58.25	13,677	4836
ASD Level 3	Communication (CI score)	28.60	9288	2937	25.50	6381	2018
	Daily life skills (CI score)	46.00	11,879	3756	39.50	9443	2986
	Socialization (CI score)	32.60	8884	2810	27.90	5259	1663
	Motor skills (CI score)	62.33	4619	2667	59.33	7371	4256
	VIN18_ABC_(total ci score)	39.90	10,682	3378	34.80	8025	2538

**Table A3:** Intervention Protocol.

Session Phases	Description of the Phase
Before the session: anticipation	The child is prepared in class for attending the sensory stimulation room. To do this, a specific stimulus is presented that the child associates with that activity. This stimulus helps the child locate themselves in time and space. Following the DSM-5 classification, the stimuli vary from oral language for Level 1 students, the use of pictograms for Level 2 students, and auditory or olfactory stimuli for Level 3 students.
The child is accompanied to the sensory stimulation room	Once in the room, each user’s access time must be respected. In the case of users with Level 3 autism, several sessions may be needed before access to the room is achieved. This may be because their information processing and expected response can, in some cases, take even several days.
The lights are turned on upon entering	We are referring to a neutral white light that the user must identify with the beginning and the end of the session.
We choose a place in the classroom for the opening ritual of the session	This place must always be the same. The user removes their shoes, and on a panel in front of them, the work plan is presented using pictograms, adjusting the communication channels available according to their needs. In the case of Level 1 and 2 users, we use clear, concrete, and precise oral language, while maintaining pictographic support. In the case of Level 3 users, we indicate what the session will consist of by guiding the user’s finger to point at the sequence.
Record keeping	A record of the sessions must be kept, either manually or digitally. The record must be completed before (psychophysiological variables), during (the user’s adaptive responses to the stimulation provided), and after the session (psychophysiological variables).
Before starting the session	Psychophysiological constants are measured: heart rate and blood oxygen level. The data are entered into the general session record.
The lights are turned off to begin the session	Interventions are grouped into three main categories following the DSM-5 classification of users with ASD. For Level 3 users, vestibular, proprioceptive, and tactile stimulation are prioritized (those at the base of the developmental pyramid, without which other skills and abilities cannot be achieved). For Level 2 users, these types of stimulation are maintained, but the intervention is enriched with activities involving the other senses as well (sight, hearing, smell, and taste). Finally, for Level 1 students, stimulation activities that promote the development of thinking and reasoning are included.
To end the session	All stimuli are removed. This is how the user understands that the session has ended. The neutral light is turned on, and the user is guided to the corner designated for the beginning and end of the session.
End of the session	The user puts their shoes back on, psychophysiological measurements are taken again, and they are informed that they are returning to class to continue with the school day.

**Table A4:** Description of the Use of Room Elements According to the Level of Autism Based on DSM-5 Classification: How It Is Used with Each Type of Student.

ELEMENT	Level 3	Level 2	Level 1
Bubble column	Tactile: the tube vibration is used to bring different body parts closer, allowing varied sensory perceptions that improve body schema understanding.	Visual: eye-hand coordination. Head tracking followed by eye-only tracking. Light games creating sequences that must be repeated. The goal is to improve attention and memory. Alternated with tactile stimulation.	Visual: working on gaze, visual tracking, and perception to improve attention. Complex color sequences are created for repetition to train memory.
Water bed	Vestibular: facilitate access with gentle rocking. Gradually vary intensities and establish non-verbal communication. Proprioception: apply pressure along limbs to improve body awareness.	Vestibular: also used to lower high arousal levels to improve attention in other activities. Proprioception: passive fall exercises combined with progressive limb pressure to reduce echolalia and encourage verbal requests.	Vestibular and proprioceptive: sequences are established to promote relaxation, improve session engagement, reduce tension, enhance concentration, reduce fatigue, and improve psycho-physical balance.
Ball pit	Alternating throws with progression. Proprioceptive and tactile. Move the ball across body parts while verbalizing.	Auditory and proprioceptive: noise and silence through interaction with the balls, improving movement self-control.	Eye-hand coordination: placing balls in a bucket or throwing them against a wall and catching rebounds by color or randomly. Eye-foot coordination: throwing balls and stopping them with the foot.
Aromatherapy set	Not applicable	Taste and smell: discrimination of aromas, association with foods, and classification of flavors. Aims to improve tolerance to different textures.	Taste and smell: contrasting essences (relaxing like rose, stimulating like mint), hot/cold foods, and sweet, salty, sour, bitter flavors.
Light fibers	Tactile: used to provide different sensations (soft, intense), wrapping around limbs to improve mental representation and encourage communication.	Visual and tactile: used to work on mathematical concepts (more/less, addition, subtraction) and imagination through play, enhancing symbolic representation.	Proprioceptive: varying pressure on joints, muscles, and tendons. Also used to develop fine motor skills.
Bobath ball	Vestibular: helps the child experience imbalance to improve postural control and interaction with the environment.	Vestibular: exercises using different body parts on the ball to develop posture and balance, with progressive difficulty.	Not applicable
Cognitive Skills Screen	Not applicable	Concepts linked to learning units are developed to improve language, attention, and memory through discrimination, cause-effect, and figure-ground programs.	Activities to stimulate thinking, attention, memory, reasoning, especially planning and execution due to difficulties with executive functions.
